# Supplementary material for: Dynamic Survival Risk Prognostic Model and Genomic Landscape for Atypical Teratoid/Rhabdoid Tumors: A Population-Based, Real-World Study
Source: Cancers (Basel). 2024 Mar 5;16(5):1059. doi: 10.3390/cancers16051059 (PMC10930634; doi:10.3390/cancers16051059)
Supplement: Supplementary file 1 [file cancers-16-01059-s001.zip › Supplementary Material.pdf]

Supplementary Material

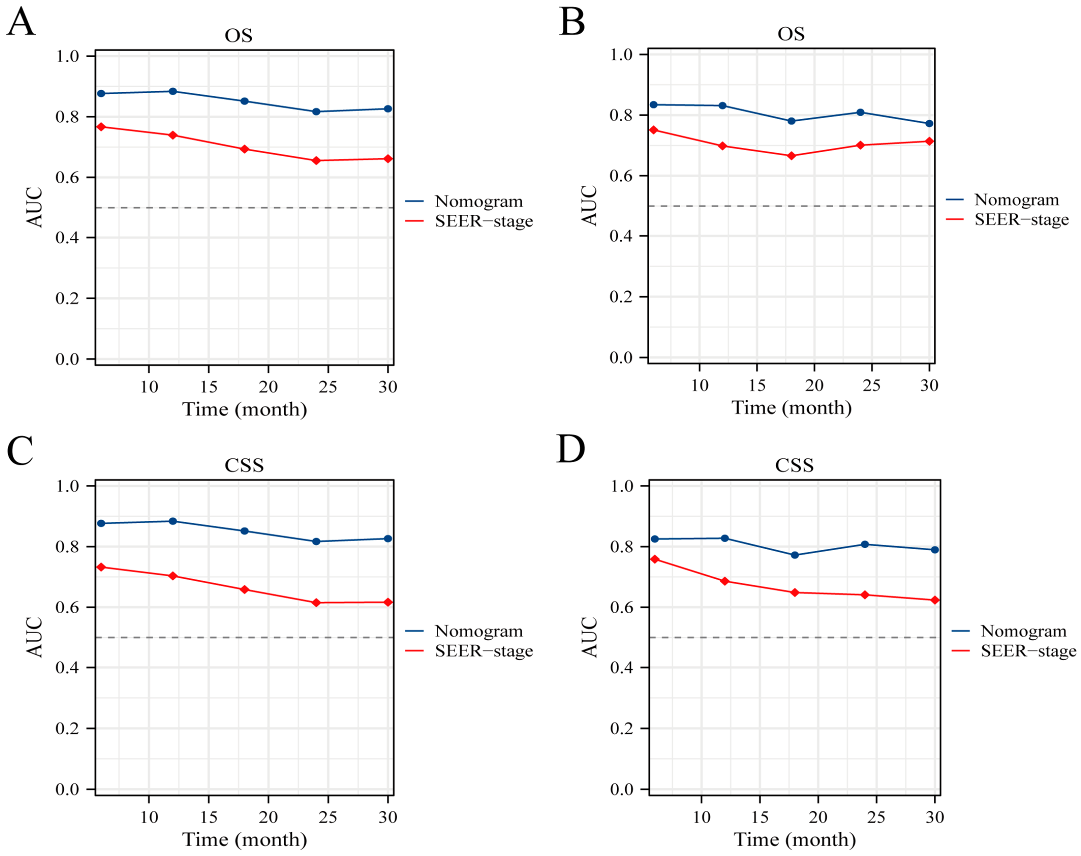

Supplementary Figure S1. Time-dependent ROC curves comparing the use of the Nomogram and SEER-stage system to predict the 1-, 2- and 3-year OS and CSS in the training cohort (A,C), the internal validation cohort (B,D).

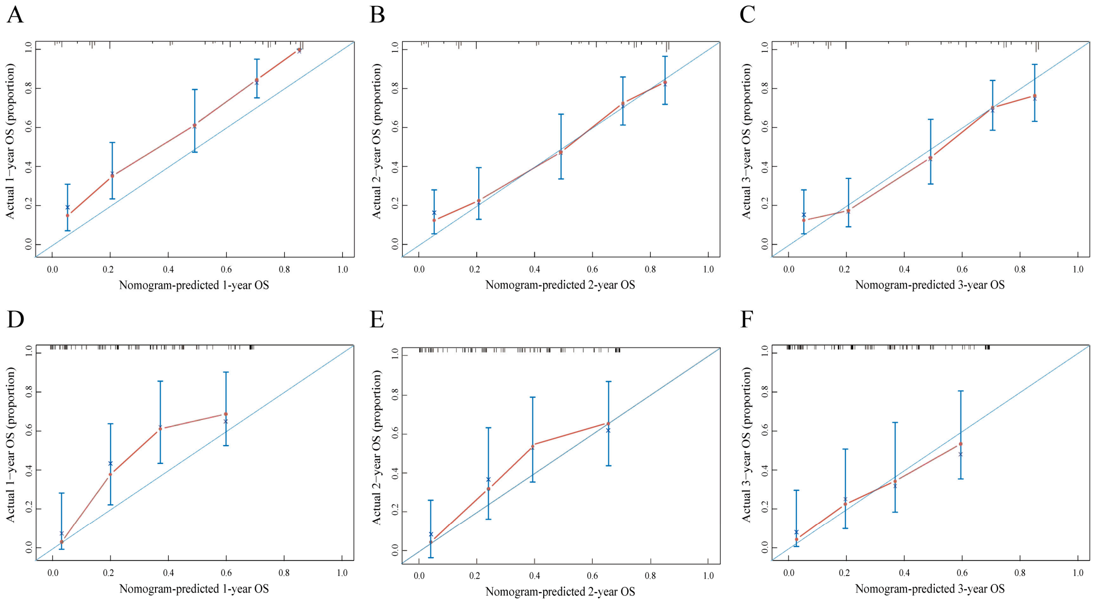

Supplementary Figure S2. The calibration curves predicting 1-year (A,D), 2-year (B,E), and 3-year (C,F) OS in training group and validation group.

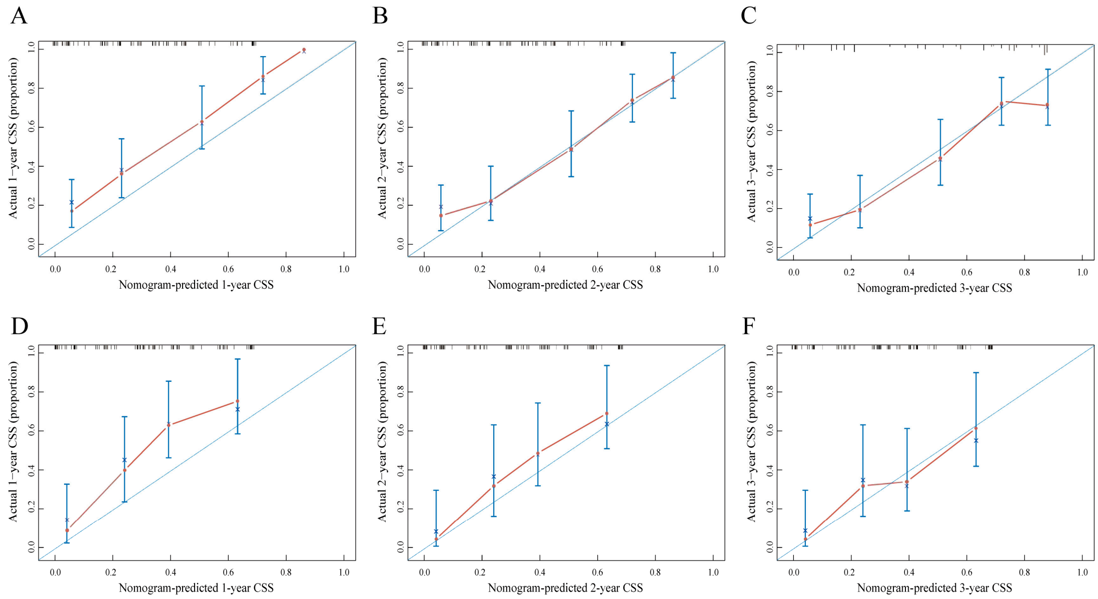

Supplementary Figure S3. The calibration curves predicting 1-year (A,D), 2-year (B,E), and 3-year (C,F) CSS in training group and validation group.

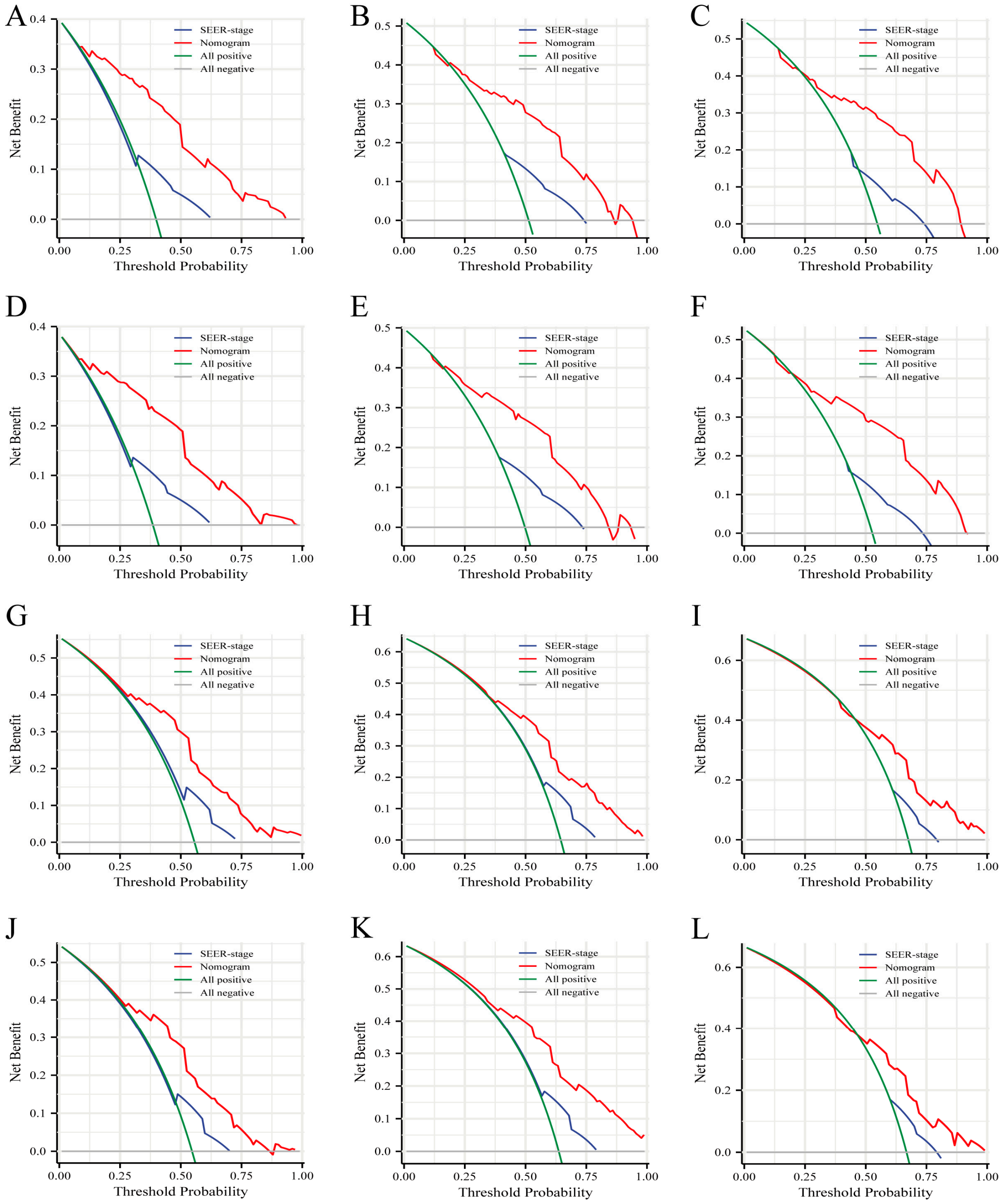

Supplementary Figure S4. The nomogram and the SEER-stage of the Decision curve analysis in the prediction of OS at the 1-year (A,D), 2-year (B,E) and 3-year (C,F) point in the training and validation groups. The prediction of CSS at the 1-year (G,J), 2-year (H,K) and 3-year (I,L) point in the training and validation groups.

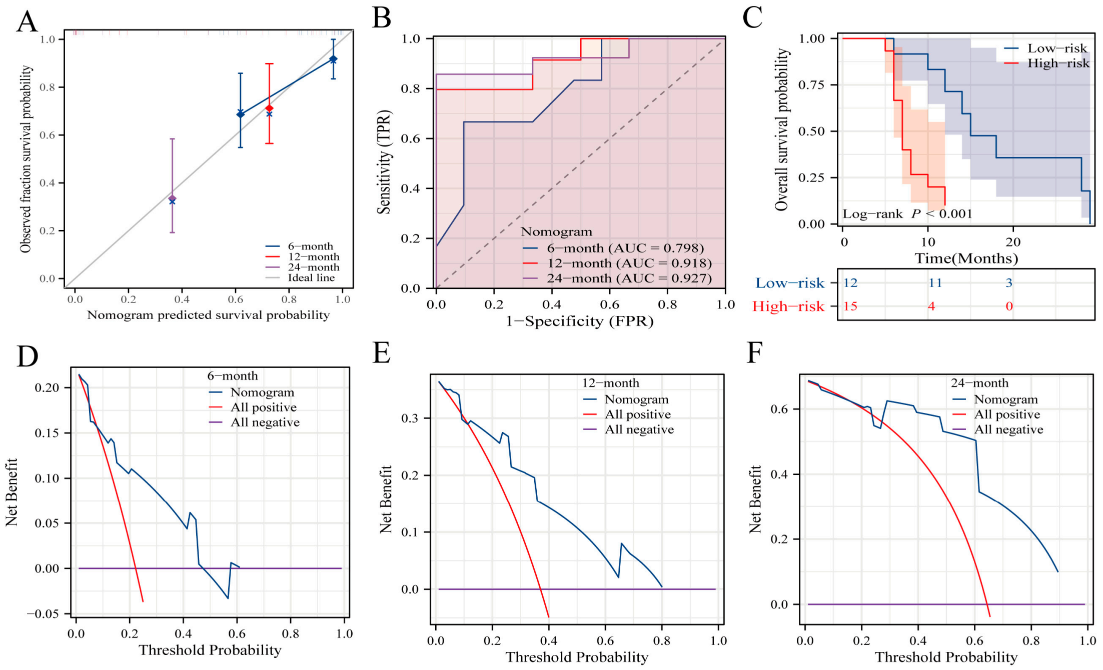

Supplementary Figure S5. The external validation of predictive models. (A) Calibration curves forecasting OS at 0.5-year, 1-year, and 2-year intervals. (B) Time-dependent ROC curve for OS. (C) Risk-stratification based on risk points derived from the model. (D-F) Decision curve analysis for OS prediction at 0.5-year (D), 1-year (E), and 2-year (F) milestones.

Supplementary Table S1. Characteristics of the AT/RT patients in the external validation group.

Characteristics	External validation group in Chongqing (n=27) no.(%)
**Median follow-up time （Months, Range）**	22 (4.3-39.7)
**Median overall survival time （Months, 95%CI）**	10 (5.8-14.2)
**Gender**	
Male	15 (55.6%)
Female	12 (44.4%)
**Age**	
<1 years	6 (22.2%)
1-3 years	15 (55.6%)
>3 years	6 (22.2%)
**Race**	
White	0 (0.0%)
Black	0 (0.0%)
Others	27 (100.0%)
**Grade**	
Poorly/Undifferentiated	20 (74.1%)
Unknown	7 (25.9%)
**Primary Site**	
Brain	21 (77.8%)
Spinal cord	6 (22.2%)
**T umor size**	
<4cm/NOS	10 (37.0%)
≥4cm	17 (63.0%)
**M stage**	
M0	11 (40.7%)
M1	16 (59.3%)
**Surgery**	
GTR/STR	25 (92.6%)
Others	2 (7.4%)
**Radiation**	
No/Unknown	23 (85.2%)
Yes	4 (14.8%)
**Chemotherapy**	
No/Unknown	13 (48.1%)
Yes	14 (51.9%)

Supplementary Table S2. Propensity-matched score analysis of triple-therapy (SRC) with surgery plus radiotherapy/chemotherapy (SB/SC) in AT/RT.

Characteristics	Unmatched			Matched		
	SR/SC N=124	SRC N=133	*P value*	SR/SC N=124	SRC N=77	*P value*
**Years of diagnosis**			<0.001			0.113
2000-2009	58 (47%)	93 (70%)		66 (53%)	32 (42%)	
2010-2019	66 (53%)	40 (30%)		58 (47%)	45 (58%)	
**Age**			<0.001			0.172
<1 year	56 (45%)	25 (19%)		56 (45%)	25(32%)	
1-3 year	52 (42%)	65 (49%)		52 (42%)	42 (55%)	
>3 year	16 (13%)	43 (32%)		16 (13%)	10 (13%)	
**Gender**			0.776			0.685
Male	64 (52%)	71 (53%)		64 (52%)	42 (55%)	
Female	60 (48%)	62 (47%)		60 (48%)	35 (45%)	
**Race**			0.478			0.893
White	93 (75%)	108 (81%)		93 (75%)	60 (78%)	
Black	18 (15%)	14 (11%)		18 (15%)	10 (13%)	
Others	13 (10%)	11 (8.3%)		13 (10%)	7 (9.1%)	
**Household income**			0.568			0.213
<75000$	88 (71%)	90 (68%)		88 (71%)	48 (62%)	
≥75000$	36 (29%)	43 (32%)		36 (29%)	29 (38%)	
**Grade**			0.529			0.982
Unknown	100 (81%)	103 (77%)		100 (81%)	62 (81%)	
III-IV	24 (19%)	30 (23%)		24 (19%)	15 (19%)	
**Primary site**			0.678			0.911
Intracranial	118 (95%)	125 (94%)		118 (95%)	73 (95%)	
Spinal cord	6 (4.8%)	8 (6.0%)		6 (4.8%)	4 (5.2%)	
**Laterality**			0.012			0.175
Left	21 (17%)	22 (17%)		21 (17%)	13 (17%)	
Right	14 (11%)	34 (26%)		14 (11%)	16 (21%)	
Others	89 (72%)	77 (58%)		89 (72%)	48 (62%)	
**Tumor size**			0.045			0.461
<4/NOS	61 (49%)	82 (62%)		61 (49%)	42 (55%)	
≥4cm	63 (51%)	51 (38%)		63 (51%)	35 (45%)	
**SEER-stage**			0.009			0.163
Localized	65 (52%)	93 (70%)		65 (52%)	50 (65%)	
Regional	23 (19%)	20 (15%)		23 (19%)	13 (17%)	
Distant	36 (29%)	20 (15%)		36 (29%)	14 (18%)	
